# Comparative studies on single-layer reduced graphene oxide films obtained by electrochemical reduction and hydrazine vapor reduction

**DOI:** 10.1186/1556-276X-7-161

**Published:** 2012-02-29

**Authors:** Zhijuan Wang, Shixin Wu, Juan Zhang, Peng Chen, Guocheng Yang, Xiaozhu Zhou, Qichun Zhang, Qingyu Yan, Hua Zhang

**Affiliations:** 1 School of Materials Science and Engineering, Nanyang Technological University, 50 Nanyang Avenue, Singapore 639798, Singaporea; 2 Center for Biomimetic Sensor Science, Nanyang Technological University, 50 Nanyang Drive, Singapore 637553, Singapore; 3 School of Chemistry and Life Science, Changchun University of Technology, 2055 Yan'an Street, Changchun, Jilin 130012, People's Republic of China

## Abstract

The comparison between two kinds of single-layer reduced graphene oxide (rGO) sheets, obtained by reduction of graphene oxide (GO) with the electrochemical method and hydrazine vapor reduction, referred to as E-rGO and C-rGO, respectively, is systematically studied. Although there is no morphology difference between the E-rGO and C-rGO films adsorbed on solid substrates observed by AFM, the reduction process to obtain the E-rGO and C-rGO films is quite different. In the hydrazine vapor reduction, the nitrogen element is incorporated into the obtained C-rGO film, while no additional element is introduced to the E-rGO film during the electrochemical reduction. Moreover, Raman spectra show that the electrochemical method is more effective than the hydrazine vapor reduction method to reduce the GO films. In addition, E-rGO shows better electrocatalysis towards dopamine than does C-rGO. This study is helpful for researchers to understand these two different reduction methods and choose a suitable one to reduce GO based on their experimental requirements.

## Introduction

Graphene, a kind of two-dimensional carbon materials, has attracted increasing attention [1-3]. Recent studies proved that the graphene-related materials have excellent characteristics in various applications such as synthesis of hybrid materials [4-11], capacitors [12,13], sensors [11,14-20], electric devices [21-24], solar cells [25-27], drug delivery [28,29], cell imaging [29], and cell cultures [30], etc. In order to produce large amount of graphene-related materials, the chemical methods are generally used [31-33].

Normally, graphene oxide (GO) is synthesized by the modified Hummers method [31-33], which is a strong oxidation method by using the concentrated sulfuric acid and potassium permanganate as oxidation agents. During the oxidation, the π-π electronic conjugation of graphite is destroyed, and the carbon sheets are decorated with the epoxide and hydroxyl groups in their basal planes as well as the carbonyl and carboxyl groups at their edges [34-36]. These functional groups render GO hydrophilic, facilitating the dispersion of GO in aqueous solution. After the reduction of GO with hydroquinone [37], NaBH_4 _[38], hydrazine hydrate [39], hydrazine vapor [31,40-43], or hydrazine with NH_3 _[44], GO is deoxygenated and reduced GO (rGO] is obtained. However, these reducing agents, particularly hydrazine, are toxic, and the use of them should be with extreme care and minimized [45]. In addition, the excessive reducing agents might contaminate the resulting product, i.e. rGO.

Recently, our group [46] and other groups [47-49] independently developed an electrochemical method to reduce GO, which is simple, fast, and green, as compared with the aforementioned chemical methods. More importantly, the highly negative potential used in the electrochemical method can overcome the energy barriers to efficiently reduce the oxygen-containing functional groups in GO [47].

Both the electrochemical reduction [20,46-49] and hydrazine vapor reduction [31,40-43,50] can reduce GO to get electrochemically reduced GO (E-rGO) and chemically reduced GO (C-rGO), referred to as E-rGO and C-rGO, respectively. In this work, after a systematic study, we found that although the morphologies of single-layer E-rGO and C-rGO adsorbed on solid substrates are quite similar, their components and especially their electrocatalysis towards dopamine (DA) are quite different. Our experiment results showed that the amount of oxygen-containing functional groups in C-rGO is more than that in E-rGO, and the electrocatalysis of E-rGO towards DA is better than that of C-rGO.

## Experimental details

Nature graphite was purchased from Bay Carbon (Bay City, Michigan, USA) and used for synthesizing GO. 3-Aminopropyltriethoxysilane (APTES), H_2_O_2 _(30%), H_2_SO_4 _(98%), phosphate buffered saline, K_4_[Fe(CN)_6_] (99.9%), K_3_[Fe(CN)_6_] (99%), hexaammine ruthenium (III) chloride (98%), dopamine hydrochloride (99%), uric acid (UA) (≥ 99%), and hydrazine hydrate (98%) were purchased from Sigma-Aldrich (Milwaukee, WI) and used as received. Ascorbic acid (AA) (≥ 99.5%, Fluka, Sigma-Aldrich), HCl (37%, Merck, Darmstadt, Germany), NH_3_·H_2_O (28%, J. T. Baker, Phillipsburg, NJ, USA), NaCl (99.5%, Merck, Darmstadt, Germany) were used as received. Toluene was purified from a solvent purification system (PS-400-5, innovative technology Inc, USA). Indium tin oxide (ITO) (10 ohm/sq, thickness, 0.7 mm) was purchased from Kintec Company (Hong Kong, China). Ultrapure Milli-Q water (Milli-Q System, Millipore, Billerica, MA, USA) was used in all experiments.

GO was synthesized based on our previous report [31]. The obtained GO powder was dispersed in water with a certain concentration by sonication to get GO aqueous solution. The adsorptions of single-layer GO on APTES-modified glassy carbon electrode (GCE) [46], ITO [46], and SiO_2 _substrates [31,50], referred to as GCE-APTES-GO, ITO-APTES-GO, and SiO_2_-APTES-GO, respectively, were prepared based on our previous reports. The obtained GCE-APTES-GO and ITO-APTES-GO electrodes were scanned in 0.5 M NaCl solution saturated with N_2 _from 0.7 to -1.1 V at a scan rate of 50 mV s^-1 ^[46].

The GCE-APTES-GO, ITO-APTES-GO, and SiO_2_-APTES-GO electrodes were reduced by hydrazine vapor at 80°C overnight based on our previous report [31].

Atomic force microscopy (AFM) images were obtained by using Dimension 3100 (Veeco, CA, USA) in tapping mode using Si tip (Veeco, resonant frequency, 320 kHz, Spring constant, 42 N m^-1^) under ambient conditions with a scanning rate of 1 Hz and scanning line of 512.

Raman spectra were recorded on a WITec CRM200 confocal Raman microscopy system using 633 nm laser with an air cooling charge-coupled device as detector (WITec Instrument Corp, Germany). Before measurement, the instrument was calibrated by silicon wafer.

All electrochemical measurements were carried out in a conventional three-electrode cell using CHI 660C Electrochemical Workstation (CHI instrument, Austin, TX, USA). In the experiment, GCE or ITO, platinum electrode, and Ag/AgCl (saturated KCl) electrode were used as working, counter, and reference electrodes, respectively. All the potentials shown in this work refer to the Ag/AgCl (saturated KCl) electrode.

## Results and discussion

**Figure 1:**
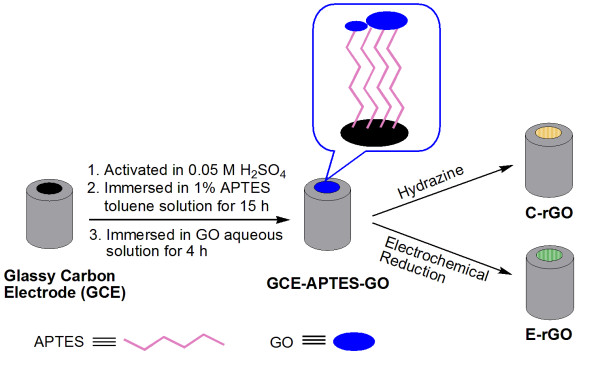
**Schematic illustration of preparation of E-rGO and C-rGO**.

The experiment process is shown in Figure 1. The single-layer GO film was adsorbed on the 3-aminopropyltriethoxysilane (APTES)-modified GCE, referred to as GCE-APTES-GO [20,46]. After GCE-APTES-GO was reduced by the electrochemical method [20,46] and hydrazine vapor reduction [31], the obtained products are referred to as E-rGO and C-rGO, respectively. As proof-of-concept, DA was chosen to compare the electrocatalysis properties of E-rGO and C-rGO.

In order to study the GO and rGO films by AFM and Raman spectroscopy, single-layer GO was assembled on APTES-modified ITO and SiO_2 _substrates, referred to as ITO-APTES-GO and SiO_2_-APTES-GO, respectively, which then was reduced by electrochemical method and hydrazine vapor reduction (see the Experimental details). Figure S1 (see Additional file 1) shows AFM images of GO and rGO films. Although a few overlaps between single-layer GO or rGO films showed up, the morphologies of the GO films before and after reduction have not shown much difference.

The previous study reported that that new C-N bond appeared in C-rGO [39]. But after the electrochemical reduction, no additional element was incorporated into the product of E-rGO [20,46]. We believe that this difference arises from the different reduction methods.

The mechanism of hydrazine vapor reduction of the functional groups, e.g. ketone, in GO might follow the probable route as shown in Figure S2 (See Additional file 1) [51], which is different from the mechanism of the electrochemical reduction of GO proposed by Dong et al. who suggested that the hydrogen ions played an essential role in the electro-reduction process [48]. In the process of hydrazine vapor reduction, the new C-N bond was introduced. But during the electrochemical reduction, the aromatic ketone, alcohol, benzylic alcohol, phenol, and aromatic carboxylic acid groups in GO might be reduced by the electrons from the extra power [52], and no new element was incorporated since no reducing agent, e.g. hydrazine, was used.

**Figure 2:**
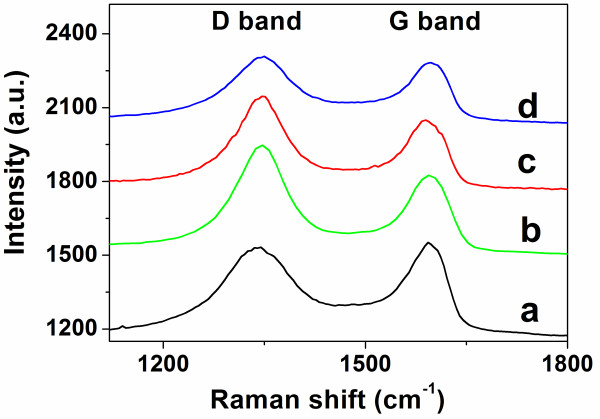
**Raman spectra of ITO-APTES-GO and C-rGO and fresh reduction of ITO-APTES-E-rGO**. Raman spectra of (a) ITO-APTES-GO, (b) ITO-APTES-C-rGO, (c) fresh reduction of ITO-APTES-E-rGO, and (d) ITO-APTES-E-rGO after kept in air for 1 day. Laser, 633 nm.

Raman spectroscopy is an effective tool to characterize the structural change of GO after reduction. In Figure 2, the peak at around 1,600 cm^-1 ^is assigned to the E_2g _mode, i.e. G band [39], and the peak at 1,350 cm^-1 ^is corresponding to the D band [38]. Both GO and rGO have these two bands. But the intensity ratio of D/G increased after the reduction (curves b, c, and d in Figure 2), suggesting a decrease in the average size of the in-plane sp^2 ^domains upon the reduction of the exfoliated GO and rGO was obtained [39]. Importantly, it should be noted that the G band shifted during the Raman measurement of E-rGO (curves c and d). For the fresh reduction product of E-rGO, it is found that G band shifted from 1,600 to 1,587 cm^-1 ^(curve c) compared with GO. But after E-rGO has been kept in air for 1 day, the G band shifted back to 1,599 cm^-1 ^(curve d). This phenomenon was not observed on the C-rGO.

Unlike the hydrazine reduction, no extra element was introduced into E-rGO during the process of electrochemical reduction. The Raman spectroscopy of E-rGO is similar to the pristine graphene, which results in the G band shift to 1,587 cm^-1^. But after being kept in air for 1 day, E-rGO might be doped by the moisture or physisorbed oxygen [53]. This p-doping was reflected by the upshift of G band [53]. In order to reduce the effect of p-doping to E-rGO, the freshly prepared E-rGO was immediately used for the following experiments.

**Figure 3:**
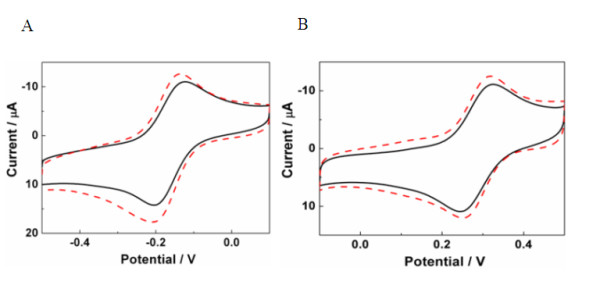
**CV curves of C-rGO (solid line) and E-rGO (dashed line)**. In (**A**) 1.0 mM [Ru(NH_3_)^6^]^3+/2+ ^(1:1) containing 0.1 M KCl and (**B**) 5 mM [Fe(CN)_6_]^3-/4- ^(1:1) containing 0.1 M KCl.

In order to evaluate the charge transfer properties of E-rGO and C-rGO, cyclic voltammograms (CV) were performed in [Ru(NH_3_)_6_]^3+/2+ ^solution (Figure 3A). The peak-to-peak separations (ΔEp) of C-rGO (solid line) and E-rGO (dashed line) are 82 and 78 mV, respectively. In addition, the difference between peak current of E-rGO and C-rGO is approximately 6 μA. This result indicates that the surface negative charge on the surface of E-rGO and C-rGO has similar effects on the electron transfer property of redox system [Ru(NH_3_)_6_]^3+/2+^. Since the GO films used for preparation of C-rGO and E-rGO are the same, the current instead of current density was used here to compare the conductivity of C-rGO and E-rGO.

To probe negative charge amount present on the C-rGO and E-rGO surface, the C-rGO and E-rGO were evaluated by CV using [Fe(CN)_6_]^3-/4- ^as indicators (Figure 3B). It has been reported that the voltammetry of [Fe(CN)_6_]^3-/4- ^is sensitive to the surface functional groups, such as hydrogen and oxygen-containing groups, present in the carbon-based electrode [54]. Therefore, in our experiment, [Fe(CN)_6_]^3-/4- ^was chosen as an indicator to explore the properties of E-rGO and C-rGO. Figure 3B represents the typical CV curves of C-rGO (solid line) and E-rGO (dashed line) in 5 mM [Fe(CN)_6_]^3-/4- ^solution containing 0.1 M KCl. The ΔE_p _of C-rGO and E-rGO are 82 and 73 mV, respectively. These ΔE_p _values indicate that the microstructural defects and density of electronic states near Fermi potential between C-rGO and E-rGO is different [54]. Since only partial functional groups of GO have been reduced, negatively charged functional groups remain on the surface of rGO. As [Fe(CN)_6_]^3-/4- ^is also negatively charged, the repulsion force between rGO and [Fe(CN)_6_]^3-/4- ^reflects the amount of the negatively charged functional groups in rGO. From Figure 3B, it can be seen that the currents for C-rGO and E-rGO are 19 and 23 μA, respectively. Therefore, the amount of negatively charged groups in C-rGO is higher than those in E-rGO, confirming that the efficiency of hydrazine vapor reduction is lower than that of electrochemical reduction.

**Figure 4:**
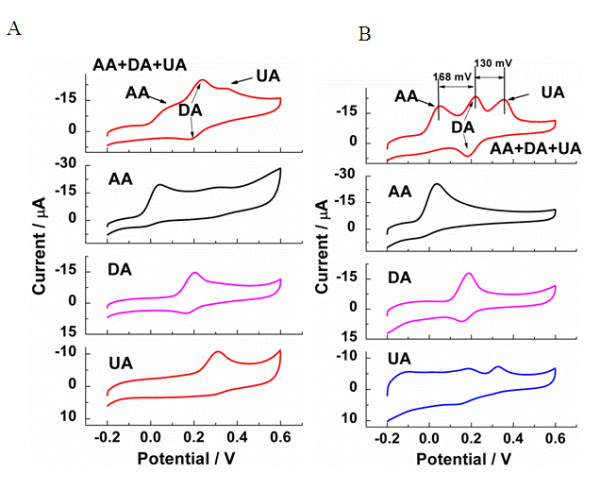
**CV curves of C-rGO and E-rGO in the mixture of AA, DA, and UA**. CV curves of (**A**) C-rGO and (**B**) E-rGO in (a) the mixture of AA (1.0 mM), DA (0.1 mM), and UA (0.1 mM), (b) 1.0 mM AA, (c) 0.1 mM DA, and (d) 0.1 mM UA. Electrolyte, 0.01 M PBS buffer (pH 7.4).

In order to compare the electrocatalytic activity of C-rGO and E-rGO, the detection of DA was performed by using C-rGO (Figure 4A) and E-rGO (Figure 4B), respectively. Usually, two factors are considered in the detection of DA. One is the interference from other electroactive species such as AA and UA [54]. Both AA and UA can be electrochemically oxidized at the potential close to DA, resulting in an overlapped oxidation peak. The other is that the concentration of AA is higher than that of DA in the neuronal regions [54]. In the pure AA, DA, or UA solution, the similar peaks were observed at C-rGO (Figure 4A) and E-rGO (Figure 4B). Such electrocatalysis of E-rGO and C-rGO towards DA is probably due to the edge-plane-like defects on rGO surface that might provide many active sites and accelerate electron transfer between the electrode and species in solution [55]. However, in the mixture of AA, DA, and UA, the obtained results are quite different. With E-rGO, well-defined oxidation peaks for AA, DA, and UA located at 52, 220 and 350 mV, respectively, showed up (Figure 4B), indicating the independently electrochemical oxidation of each analyte. However, with C-rGO, the oxidation peaks for the mixed analytes did not separate well (Figure 4A). The oxygen-containing species present on the graphene sheets are responsible for the electron transfer as well as the adsorption/desorption of molecules [56]. The better electrocatalytic activity of E-rGO might arise from the smaller amount of oxygen functional groups left on the surface of E-rGO. Therefore, E-rGO, instead of C-rGO, was applied to detect DA in the mixture solution in the following experiment.

**Figure 5:**
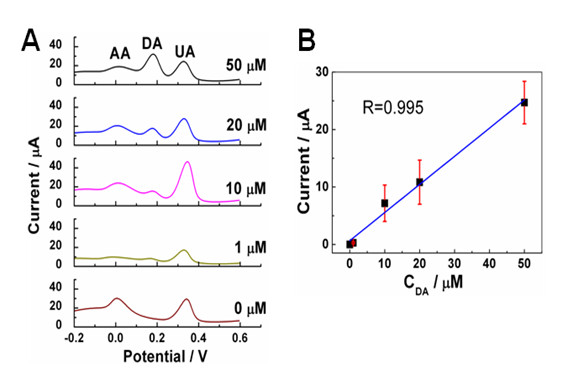
**DPV profiles of E-rGO and the plot of DPV peak as function of DA concentration**. (**A**) DPV profiles of E-rGO in the solution of 0.01 M PBS (pH 7.4) containing 1 mM AA, 0.1 mM UA, and different concentration of DA (from 0 to 50 μM). (**B**) The plot of DPV peak current as function of DA concentration.

Using the differential pulse voltammetric (DPV) technique, DA was detected with E-rGO (Figure 5). The peak intensity of DA increased with the concentration of DA (Figure 5A). Note that the DPV peak current is linear to the concentration of DA in the range of 0 to 50 μM (Figure 5B). Even at the very low concentration of DA, e.g. 1 μM, DA can be detected from the mixture of 1 mM AA, 0.1 mM UA, and 1 μM DA.

## Conclusions

Single-layer rGO, obtained by electrochemical method (referred to as E-rGO) and hydrazine vapor reduction (referred to as C-rGO), respectively, was compared in morphology and the component change after reduction and electrocatalysis towards DA. In the hydrazine vapor reduction, nitrogen was incorporated into the product, while no additional element was introduced during the electrochemical reduction. After reduction, the morphology has not shown obvious difference between the single-layer GO and rGO films. However, the CV results indicate that the electrochemical method is more effective to reduce GO films than does the hydrazine vapor reduction method. In addition, the product E-rGO showed better electrocatalysis towards dopamine than does C-rGO. Our comparative study is helpful for researchers to understand these two reduction methods and choose a suitable one to reduce GO based on their experimental requirements.

## Competing interests

The authors declare that they have no competing interests.

## Authors' contributions

HZ led the project, participated in the design of the experiments, analysis of the data and revision of the manuscript. ZW designed and carried out the experiments, and drafted the manuscript. SW and JZ carried out some electrochemical experiments. PC participated in the Raman measurement. XZ helped the Raman analysis. GY did the XPS measurements. QZ and QY revised the manuscript. All authors read and the final manuscript.

## Supplementary Material

Additional file 1 **Figure S1 and Figure S2**. For Figure S1, AFM images of (A) ITO-APTES-GO, (B) ITO-APTES-E-rGO, (C) SiO_2_-APTES-GO and (D) SiO_2_-APTES-C-rGO. For Figure S2, proposed mechanism for reduction of ketone in GO by hydrazine vapor [2]. Click here for file
