# Supplementary material for: Comparative studies on single-layer reduced graphene oxide films obtained by electrochemical reduction and hydrazine vapor reduction
Source: Nanoscale Res Lett. 2012 Feb 29;7(1):161. doi: 10.1186/1556-276X-7-161 (PMC3309976; doi:10.1186/1556-276X-7-161)
Supplement: Additional file 1 — Figure S1 and Figure S2. For Figure S1, AFM images of (A) ITO-APTES-GO, (B) ITO-APTES-E-rGO, (C) SiO2-APTES-GO and (D) SiO2-APTES-C-rGO. For Figure S2, proposed mechanism for reduction of ketone in GO by hydrazine vapor [2]. [file 1556-276X-7-161-S1.DOCX]

Supporting information

After GO was adsorbed on APTES-modified ITO and SiO_2_ substrates, referred to as ITO-APTES-GO and SiO_2_-APTES-GO, respectively, they were reduced by the electrochemical method and hydrazine vapor, and the products, referred to as ITO-APTES-E-rGO and SiO_2_-APTES-C-rGO, respectively, are obtained. AFM was used to characterize the morphologies of the films (Figure S1), showing that the single-layer GO was successfully adsorbed on substrates (Figure S1A and C) and the reduced products, i.e. E-rGO and C-rGO sheets, still existed on the substrates (Figure S1B and D). These results are consistent with our previous results [1].

**
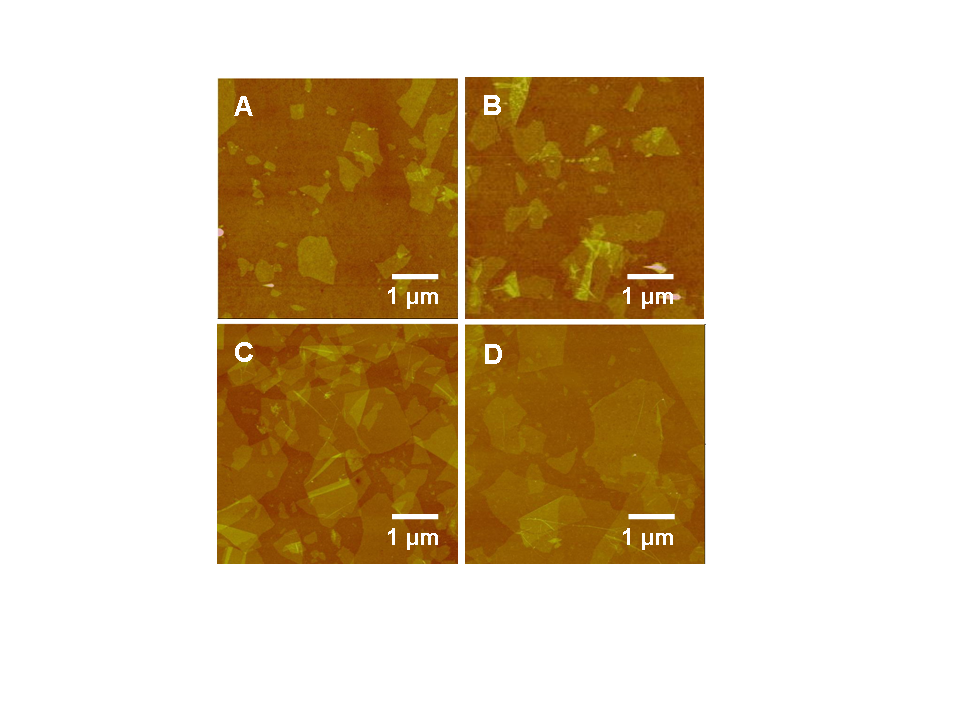
**

**Figure S1.** AFM images of (A) ITO-APTES-GO, (B) ITO-APTES-E-rGO, (C) SiO_2_-APTES-GO and (D) SiO_2_-APTES-C-rGO.

The mechanism of hydrazine vapor reduction of the functional groups, *e.g.* ketone, in GO might follow the probable route as shown in Figure S2. A Subsequent proton capture at the carbon terminal takes place in hydrazine-water vapor via a concerted fashion, producing a diimide (C). After that, a base-induced loss of dinitrogen occurs to afford carbon anion (D), which undergoes the protonation to provide the decarbonylation product and a base [2].


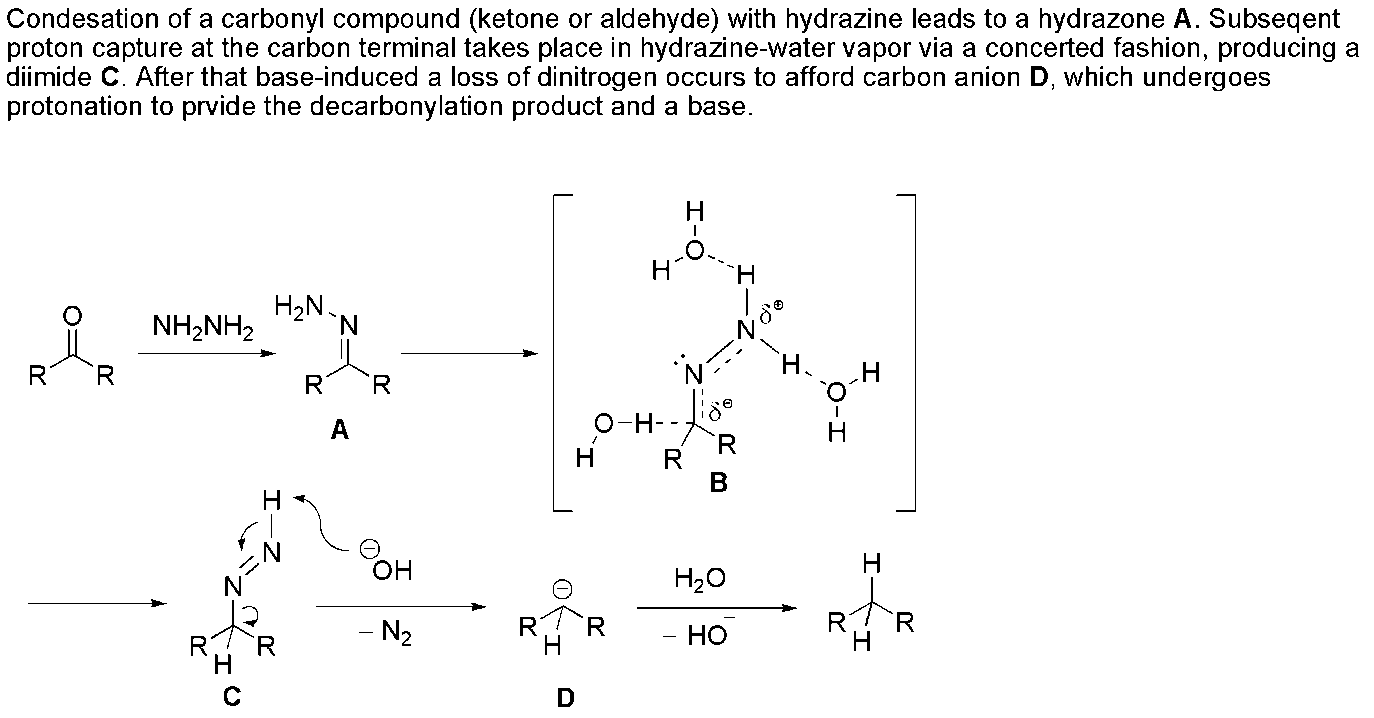


**Figure S2.** Proposed mechanism for reduction of ketone in GO by hydrazine vapor [2].

**References**

1. Wang ZJ, Zhou XZ, Zhang J, Boey F, Zhang H: **Direct electrochemical reduction of single-layer graphene oxide and subsequent functionalization with glucose oxidase.** *J Phys Chem C* 2009, **113:**14071.
2. Lάszlό K, Czakό B: In *Strategic Applications of Named Reactions in Organic Synthesis.* Elsevier Academic Press; 2005:496.
